# Invasive *Mycobacterium chimaera* Infections and Heater–Cooler Devices in Cardiac Surgery

**DOI:** 10.3201/eid2603.180452

**Published:** 2020-03

**Authors:** Theresa L. Lamagni, André Charlett, Nick Phin, Maria Zambon, Meera Chand

**Affiliations:** Public Health England, London, UK (T.L. Lamagni, A. Charlett, N. Phin, M. Zambon, M. Chand);; Imperial College, London (M. Zambon, M. Chand);; Guy’s & St. Thomas’ NHS Foundation Trust, London (M. Chand)

**Keywords:** nontuberculous mycobacteria, tuberculosis and other mycobacteria, equipment contamination, disease outbreaks, cardiac surgical procedures, risk assessment

**To the Editor**: In their recent assessment of *Mycobacterium chimaera* risk in patients undergoing heart valve surgery, Sommerstein et al. compare their findings to our prior risk assessment for UK patients (*1*,*2*). In their article, the authors note their assessed risk as “4 to 7” times higher than our risk estimate and suggest this relates to differences in case-finding methodology. Our study reported incidence density (cases per 10,000 person-years) to account for the differing lengths of postoperative follow-up in each successive annual cohort of surgical patients. In contrast, Sommerstein et al. calculated crude risk based on annual procedure numbers. Since our published assessment was undertaken some years before the authors’ assessment, additional cases have been diagnosed, in keeping with the long incubation period for these infections, a median of 15 months but up to 5 years (*3*). Recalculation of risk and 95% (binomial) CIs, limited to 2008–2014 to match the authors’ assessment, would yield a crude risk estimate of 0.24 (0.15–0.35) per 1,000 procedures (24/102,234); the risk in Switzerland (11/14,054) would be estimated at 0.78 (0.39–1.40), just over 3 times higher.

Whether the observed differences between the United Kingdom and Switzerland represent a true difference in *M. chimaera* risk in patients undergoing heart valve surgery is subject to debate. Both countries based case finding on results from routine diagnostic investigation; however, awareness of the risk in Switzerland predates that in other countries, potentially increasing the likelihood of investigation for mycobacterial infection. We have observed considerable variation in risk between cardiac centers, from 0 cases rising to 1 per 100 patients for 1 center in their year of highest estimated risk (*4*). Our pooled estimate covering 33 centers may encompass a wider selection of risk profiles, compared with the smaller number of centers in Switzerland.
